# Clinicopathological Features and Immunochemical Staining of Inflammatory Myofibroblastic Tumor: A Retrospective Study of 48 Cases

**DOI:** 10.1155/ancp/4948627

**Published:** 2025-11-18

**Authors:** Qi-An Wang, Ren-Chin Wu, Chao-Wei Lee, Yu-Hsuan Yeh, Chiao-En Wu

**Affiliations:** ^1^ School of Medicine, College of Medicine, Chang Gung University, Taoyuan, Taiwan, cgu.edu.tw; ^2^ Department of Pathology, Chang Gung Memorial Hospital Linkou Branch, Chang Gung University College of Medicine, Taoyuan, Taiwan, cgu.edu.tw; ^3^ Division of General Surgery, Department of Surgery, Chang Gung Memorial Hospital, Linkou Medical Center, Taoyuan, 333, Taiwan, cgmh.org.tw; ^4^ School of Traditional Chinese Medicine, College of Medicine, Chang Gung University, Taoyuan, Taiwan, cgu.edu.tw; ^5^ Division of Hematology-Oncology, Department of Internal Medicine, Chang Gung Memorial Hospital at Linkou, Chang Gung University College of Medicine, Taoyuan, Taiwan, cgu.edu.tw; ^6^ Division of Hematology-Oncology, Department of Internal Medicine, New Taipei Municipal TuCheng Hospital, New Taipei City, 236043, Taiwan

## Abstract

Inflammatory myofibroblastic tumors (IMTs) are rare neoplasms found in diverse anatomical sites, including the lungs, intestines, and bladder. Surgical resection is the primary treatment, with chemotherapy offering survival rates of 21.2 and 42.5 months in unresectable cases. Anaplastic lymphoma kinase (ALK) tyrosine kinase inhibitors (TKIs) have emerged as standard treatments. However, IMT presents diagnostic challenges owing to its morphological similarities with other conditions. While positive ALK expression aids diagnosis in approximately 50% of the cases, negative ALK expression complicates diagnosis. This retrospective study reviewed 48 patients with IMT diagnosed at a Taiwanese medical center and analyzed their clinical characteristics, pathology reports, immunohistochemical (IHC) staining profiles, and outcomes. Chi‐square and independent *t*‐tests were used for the statistical analysis. The results showed an equal sex distribution, with the gastrointestinal tract being the most prevalent site, followed by the urinary system and lungs. Positive ALK expression was observed in 12 patients. While no statistically significant differences were found between ALK expression and age, sex, or site of occurrence, a younger mean age was noted in the ALK‐positive group, which is consistent with the existing literature. Moreover, ALK negativity tended to occur in the nonvisceral organs (head and neck, extremities, and genitals). Regarding IHC staining, smooth muscle actin (SMA) was positive in 74.5% of cases, whereas S‐100 protein, CD34, CD117, myogenin, and DOG1 were consistently negative. For desmin and cytokeratin, the results differed among cases and may not be used as determinant factors to diagnose such diseases. We hope that this investigation will be a cornerstone for further studies on the diagnosis of IMT in the absence of ALK rearrangements.

## 1. Introduction

Inflammatory myofibroblastic tumors (IMTs) are rare tumors that occur in various anatomical locations [1]. In terms of treatment, IMT is usually treated by surgical resection followed by low rate of recurrence. For the unresectable tumors, chemotherapies could also generate acceptable outcomes with overall survival ranging from 21.2 to 42.5 months [2], along with other newly emerged treatment, such as anaplastic lymphoma kinase (ALK) tyrosine kinase inhibitors (TKIs), which have been considered as the standard treatment [1]. However, IMTs have historically posed diagnostic challenges owing to their morphological resemblance to other similar conditions, such as gastrointestinal stromal tumors (GISTs), peripheral nerve sheath tumors, or inflammatory fibroid polyps [3, 4]. Given the nonspecific nature of IMT symptoms and clinical progression, diagnosis has primarily relied on pathological features, notably the presence of proliferating fusiform spindle cells with one to three nucleoli within round nuclei [1]. The identification of ALK expression in approximately half of patients with IMT has improved the diagnostic accuracy [5], yet poses difficulties for those lacking ALK expression. Immunohistochemical (IHC) staining has become increasingly crucial for diagnosis [1], although the rarity of IMT has hindered the large‐scale cohort studies necessary for systematic findings.

Most of the existing literature is limited to a small number of patients owing to the scarcity of cases. This retrospective study aimed to explore the real‐world data of patients with IMT diagnosed at a medical center in Taiwan. An extensive analysis was conducted to assess whether different factors influenced ALK expression in patients with IMT. Additionally, by utilizing the data of patients’ IHC staining profiles, we expect to build a diagnostic framework for patients with ALK(−) IMT. The results may be useful for pathologists to decide which biomarkers to stain for if ALK expression is negative, and if the clinical traits are indistinguishable from other differential diagnoses.

## 2. Method

This retrospective investigation focused on patients diagnosed with IMT at Chang Gung Memorial Hospital (CGMH), Linkou Branch, between 2001 and 2022. The inclusion criteria encompassed individuals meeting the following conditions: (1) histopathologically confirmed diagnosis of IMT, (2) IMT occurrence at any anatomical site, (3) IMT diagnosis at any age, and (4) treatment (including surgeries, radiotherapy, chemotherapy, or targeted therapy) at CGMH, Linkou Branch. Patients were excluded if a definitive diagnosis of IMT could not be established.

Data on clinical characteristics, pathological reports, treatments, and outcomes, including age, sex, IMT localization, IHC staining profiles, and administered treatments, were collected. Due to the retrospective nature of this study and the absence of definitive IHC markers for diagnosing IMT, the selection of IHC markers relied heavily on the clinical judgment of the attending pathologist and the specific clinical scenario. Consequently, certain markers were utilized more frequently than others, reflecting variations in pathologists’ preferences and diagnostic approaches. Markers that were stained less frequently, including CD21, CD35, and CD23, are detailed in Table S1. Regarding the clones of IHC markers, those commonly stained in more than 50% of studied population were summarized in Table 1. Statistical analyses were performed using chi‐square tests and independent *t*‐tests to explore the association between ALK expression and categorical (sex and sites of occurrence) and continuous variables (age), respectively. Statistical significance was set at *p*  < 0.05.

**Table 1:** Clones of IHC antibodies commonly stained.

Marker	Clones	Location	Brand	Dilution
ALK	5A4	Cytoplasmic	Leica	100x
ALK	D5F3	Cytoplasmic	Roche	Original
SMA	1A4	Cytoplasmic	DAKO	400x
Desmin	D33	Cytoplasmic	DAKO	500x
Cytokeratin	AE1/AE3	Cytoplasmic	DAKO	500x
S‐100 protein	EP32	Cytoplasmic/nuclear	Leica	500x

Abbreviations: ALK, anaplastic lymphoma kinase; SMA, smooth muscle actin.

This retrospective study was approved by the Institutional Review Board of CGMH (IRB: 202300977B0). Owing to the retrospective nature of this study, the requirement for informed consent was waived.

## 3. Results

### 3.1. Characteristics of Patients

A total of 48 patients diagnosed with IMT were included in this analysis. The baseline characteristics of the 48 patients pathologically diagnosed with IMT are summarized in Table 2. Most patients (*n* = 41; 85.4%) were aged <65 years, with a median age of 47 years, and half of the patients (*n* = 24; 50%) were male. For IMT, the gastrointestinal tract appeared to be the most common site (15 patients; 31.2%), followed by the urinary system (10 patients; 20.8%), pulmonary region (nine patients; 18.8%), genital region (five patients; 10.4%), extremities (five patients; 10.4%), and head and neck region (four patients; 8.3%). Moreover, ALK positivity was only present in 12 patients (27.9%), and there were five patients who confirmed the diagnosis of IMT by pathological report without IHC staining. In terms of treatment, most patients underwent surgical resection (45 patients; 93.8%), while one received radiotherapy, and the other two patients received ALK‐TKIs. However, treatment outcomes were satisfactory. Most patients remained disease‐free after surgical resection, and recurrence was observed in only one patient. Residual sarcoma with peritoneal seeding was observed 1 month after the operation. Chemotherapy (vincristine, epirubicin, ifosfamide, and etoposide) was administered for the recurrent lesions; however, little efficacy was noted. The patient appeared hopeless and refused other advanced treatments; therefore, the family required discharge against medical advice. Furthermore, one patient with bladder IMT received postoperative adjuvant vesical instillation postoperatively of epirubicin and remained disease‐free for 7 months till the last date of cystoscopy examination. Of the two patients who received ALK‐TKIs, one had the tumor removed surgically after targeted therapy, while the other was progression‐free for 30 months. The patient received radiotherapy (total dosage of 1600 cGy in three fractions) as a foreign worker and was returned to the country for further management after being stabilized by radiotherapy.

**Table 2:** Basic characteristics of patients (*n* = 48).

Characteristics	Number of patients	%
Age (years)		
Median ± SD	47 ± 22.65	
≤65	41	85.4
>65	7	14.6
Sex		
Male	24	50
Female	24	50
Sites of occurrence		
Head and neck	4	8.3
Pulmonary	9	18.8
Extremities	5	10.4
Gastrointestinal tract	15	31.2
Urinary system	10	20.8
Genital region	5	10.4
IHC staining of ALK (*n* = 43)		
Positivity	12	27.9
Negativity	31	72.1
Initial treatment		
Surgery	45	93.8
Radiotherapy	1	2.1
ALK TKI	2	4.2
Recurrence	1	2.1

### 3.2. IHC Staining of IMT

The results of the diagnostic approach to IMT are summarized in Table 3. In addition to ALK expression, smooth muscle actin (SMA) was predominantly expressed (29 patients; 74.4%). However, desmin (20 patients; 52.6%), cytokeratin (19 patients; 73.1%), S‐100 protein (24 patients; 96%), CD34 (*n* = 20; 95.2%), CD117 (*n* = 18; 100%), and myogenin (*n* = 2; 100%), discovered on GIST (DOG1, *n* = 2; 100%), usually stained negatively in patients with IMT.

**Table 3:** IHC staining of patients.

Characteristics	Number of patients	%
ALK (*n* = 43)		
Positive	12	27.9
Negative	31	72.1
Smooth muscle actin (SMA, *n* = 39)		
Positive	29	74.4
Negative	10	25.6
Desmin (*n* = 38)		
Positive	18	47.4
Negative	20	52.6
Cytokeratin (CK, *n* = 26)		
Positive	7	26.9
Negative	19	73.1
S‐100 protein (*n* = 25)		
Positive	1	4
Negative	24	96
CD34 (*n* = 21)		
Positive	1	4.8
Negative	20	95.2
CD117 (*n* = 18)		
Positive	0	0
Negative	18	100
Myogenin (*n* = 2)		
Positive	0	0
Negative	2	100
DOG1 (*n* = 2)		
Positive	0	0
Negative	2	100

Abbreviations: CD, cluster of differentiation; DOG1, discovered on GIST.

In addition, the markers to be stained largely depend on the clinical judgment of the pathologist, and the resulting options for IHC vary between pathologists. Other stained IHC markers (e.g., CD21, CD35, and CD23) are summarized in Table S1.

### 3.3. Subgroup Analysis of ALK Expression

To further evaluate the relationship between ALK expression and other factors, chi‐square and independent *t*‐test were performed. No statistically significant differences were found for age (*p* value = 0.595), sex (*p* value = 0.662), or site of occurrence (*p* value = 0.064; Table 4). The relationship between ALK expression and age was studied further by stratifying the age groups into ≤18 years old, 19–64 years old, and ≥65 years old. These results were not statistically significant (*p* value = 0.242; Table 4). However, an intriguing finding has emerged. Despite the statistical insignificance between the origin of the IMT and ALK expression, by categorizing the sites of origin in the order of visceral and nonvisceral organs, we observed that ALK was expressed only in visceral IMT and not in nonvisceral IMT (Table 5).

**Table 4:** Relationship between ALK expression and factors.

Characteristics	ALK(+)	ALK(−)	*p* value
Age (years)			0.595^a^
Median ± SD	35 ± 20.21	43 ± 24.21	
Youngest	3	11 months	
Oldest	57	84	
Stratified age group			0.242^b^
≤18	3	7	
19–64	9	19	
≥65	0	5	
Sex			0.662^b^
Male	5	17	
Female	7	14	
Sites of occurrence			0.064^b^
Head and neck	0	4	
Pulmonary	2	4	
Extremities	0	5	
Gastrointestinal tract	5	8	
Urinary system	5	5	
Genitals	0	5	
Visceral organs	12	17	0.004
Nonvisceral organs	0	14	

Abbreviation: SD, standard deviation.

^a^The analysis was performed using independent *t*‐test.

^b^The analysis was performed using chi‐square.

**Table 5:** ALK expression in visceral vs. nonvisceral organs.

Sites of IMT	ALK(+)	ALK(−)	ALK not stained
Visceral organs (%)^a^	12 (41.4%)	17 (58.6)	—
Pulmonary	2	4	3
Gastrointestinal tract	5	8	2
Urinary system	5	5	0
Nonvisceral organs (%)^a^	0 (0%)	14 (100%)	—
Head and neck	0	4	0
Extremities	0	5	0
Genitals	0	5	0

^a^Percentage was calculated as: [number of ALK(+) or (−)]/(stained cases).

## 4. Discussion

This study presents real‐world data of 48 patients with IMT, along with their IHC staining profiles, predominantly including ALK, SMA, cytokeratin, desmin, and S‐100 proteins. Furthermore, the relationships between ALK and different factors (age, sex, and site of occurrence) were examined; however, the results were not statistically significant. However, after reviewing the data, we observed that ALK was exclusively expressed in IMT originating from the visceral organs (pulmonary, gastrointestinal tract, and urinary system), whereas IMT (*n* = 12) originating from nonvisceral organs (head and neck, extremities, and genitals) did not show any ALK positivity.

Demographically, it has been reported to occur in both sexes without any discernible preference and across various age groups, with a slight tendency observed in children and young adults [1]. In our study, the median age was 47 years, with an equal distribution in both sexes. Regarding the sites of occurrence, the lungs, abdominal regions, and soft tissues across the body have been predominantly reported [1]; however, this tumor has also been found in other anatomical areas, such as the spinal cord [6] or uterus [7], which was not found in our cohort. This is consistent with the results of our study, with the gastrointestinal tract being the prevailing site. Moreover, in our study, the urinary system, kidney and bladder, was the second most common sites of occurrence, with a total of 10 patients (20.8%), followed by nine patients (18.8%) with pulmonary IMT. Furthermore, in terms of prognosis, IMT is classified as a tumor with intermediate malignancy [8] owing to its low rates of metastasis and recurrence [5], which was observed in only one out of 48 patients with recurrence and no metastasis.

Within our patient population, only 27.9% of IMT were positive for ALK expression. However, this result was much lower than the evidence presented in the existing literature, stating that approximately half of the patients with IMT express ALK positivity [1, 3]. This phenomenon may be due to several factors. First, some studies have reported that ALK expression is more likely to be observed in younger patients than in adults [5]. This observation was supported by another study that included 59 patients with IMT [9]. Despite the lack of statistical significance in our analysis of the relationship between ALK expression and age, a tendency toward positive ALK expression was found in younger patients, with a mean age of 35‐year‐old in the ALK(+) group and 43‐year‐old in the ALK(−) group. Second, all ALK expression examinations in our study were performed solely using IHC staining. A few studies have suggested that fluorescence in situ hybridization (FISH) or next‐generation sequencing (NGS) may be alternatives to IHC, owing to the differences between the detected phenotypes and actual genotypes [3, 5]. In the study by Lovly et al. [10], NGS was employed to validate the outcome of IHC staining; and among 11 ALK(−) IMT, two IMT cases were identified to harbor ALK rearrangement, indicating that the potential bias was contributed by the IHC staining. Finally, we hypothesized that ALK expression is linked to IMT sites. As shown in Table 4, by categorizing the sites of origin into visceral (pulmonary, gastrointestinal tract, and urinary system) and nonvisceral (hand and neck, extremities, and genital region) organs, visceral organs tended to present a higher percentage of ALK positivity than IMT with nonvisceral organ origin, and none of the IMT from nonvisceral organs displayed a positive expression of ALK. In the study by Telugu et al. [11], by utilizing the published data, 12 cases had visceral origin (five from lung, one from common bile duct, one from suprarenal region, three from omentum, and two from colon), the other six cases had nonvisceral origin (three from head and neck region, one from breast, one from bronchus, and one from mediastinum). There were eight (66.7%) ALK(+) IMT cases from visceral organs and only two (33.3%) ALK(+) IMT cases from nonvisceral organs [11]. Yet, owing to the rarity of IMT, further cohort studies are required to verify this hypothesis.

**Figure 1:**
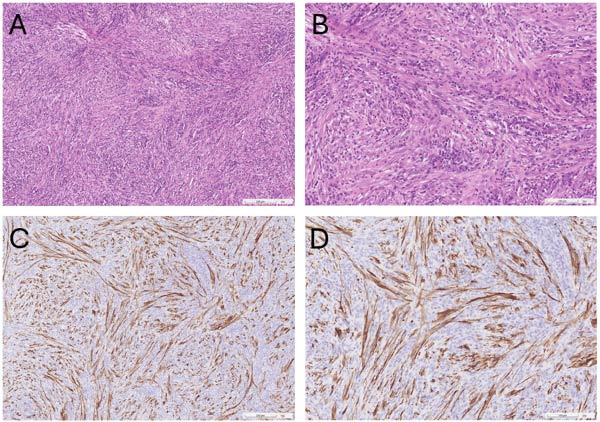
Pathological and IHC staining of IMT. (A) Hematoxylin and eosin stain (×10) and (B) hematoxylin and eosin stain (×20), revealed heavy inflammatory infiltration in a spindle cell tumor. (C) IHC stain (×10) and (D) IHC stain (×20) showed positive expression of anaplastic lymphoma kinase.

**Figure 2:**
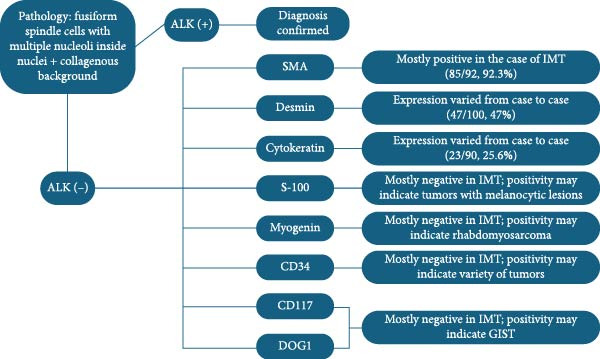
Flowchart of diagnosing IMT with IHC staining. ALK, anaplastic lymphoma kinase; CD, cluster of differentiation; DOG1, discovered on GIST; IMT, inflammatory myofibroblastic tumor; SMA, smooth muscle actin.  ^∗^The denotation among brackets was (cases of positive expression/total stained cases, percentage of positive expression).

Moreover, other than ALK, different IHC markers have been applied to differentiate diseases with similar manifestations as IMT, such as GIST or other inflammatory lesions [4], owing to the difficulty posed by the highly similar morphology [1]. Classified as an inflammatory spindle cell neoplasm [12], IMT is characterized by the proliferation of neoplastic fibroblastic and myofibroblastic cells accompanied by chronic inflammatory infiltration comprising lymphocytes, plasma cells, eosinophils, and histiocytes [3] (Figure 1). Differentiating IMT from nonneoplastic reactive lesions such as inflammatory pseudotumor (IPT) and IgG4‐related disease is essential. Unlike IMT, IPT exhibits a high recurrence rate after surgical excision, has low metastatic potential, and does not recur or metastasize following resection [12]. IgG4‐related disease, which can affect nearly every organ, is diagnosed based on the IgG4/IgG ratio; when presenting as a mass, it is termed IgG4‐related IPT [13]. IgG4‐related IPT is distinguished by a significant proliferation of IgG4‐positive plasma cells and a higher IgG4/IgG ratio (>50%). However, some research suggests that this ratio alone is not a reliable discriminator between IMT and IgG4‐related disease. The work of Saab et al. [14] revealed that while an IgG4/IgG plasma cell ratio ≥0.4 or 0.5 is often indicative of IgG4‐related sclerosing disease, some IMT cases also fall within this range, including 10 cases with ratios of 0.10–0.40 and five cases with ratios of 0.40–1.0. Careful consideration is also required to distinguish IMT from several malignant lesions. For example, inflammatory well‐differentiated liposarcoma, a rare and locally aggressive retroperitoneal tumor, is defined by overexpression of mouse double minute 2 (MDM2) homolog and cyclin‐dependent kinases 4 (CDK4), with potential progression to dedifferentiated liposarcoma [15–17]. GIST, typically found in the abdominal cavity and frequently positive for CD117 and DOG‐1, is associated with mutations in KIT or platelet‐derived growth factor receptor A (PDGFRA) [15, 18]. Desmoid fibromatosis, a locally aggressive lesion driven by β‐catenin or adenomatous polyposis coli (APC) mutations, resembles IMT, but notably lacks inflammatory infiltration [15]. Other neoplastic lesions requiring differentiation include inflammatory fibroid polyp, benign gastrointestinal neoplasms associated with PDGFRA mutations and identified by CD34(+) and CD117(−) IHC profiles [15, 19]. Nodular fasciitis, a benign, self‐limiting tumor associated with USP6 rearrangement, commonly affects the upper extremities and typically resolves spontaneously [15, 20]. Table 6 summarizes the reported expression of markers (SMA, desmin, cytokeratin, and S‐100 protein) in the literature, and the chosen markers were those that had been stained in >50% of the patient population. Furthermore, owing to the rarity of IMT and to avoid bias, we summarized the number of positive expressions and total number of stained cases from each study. Based on the table, SMA was found to be almost positively expressed, S‐100 protein was nearly always absent, and the expression of desmin and cytokeratin varied between the studies. For SMA, it was suggestive that this marker can be used to distinguish smooth muscle cells and pericytes from other cell lineages [27]. In the case of IMT, although some may argue that expression varies between cases [5], SMA is usually positively expressed [3, 28], confirming the presence of myofibroblasts within the tumor. In contrast, the S‐100 protein is typically expressed in Schwann cells, glial cells, and melanocytes, and is usually used to identify metastatic melanoma or nerve sheath tumor [27]. Therefore, it is mostly absent in the cases of IMT owing to the major components of myofibroblasts, fibroblasts, and inflammatory cells presented [1], despite the 50% positive expression in the work of Jung et al. [21]. Desmin and cytokeratin, two intermediate filament protein [29, 30], have been reported to be highly various in terms of their expression, ranging from negativity to focal or strong positivity [11, 21–26], which may imply that they were unable to act as the definitive marker to confirm the diagnosis of IMT, rather than narrowing down the differential diagnosis of similar diseases. Other markers, such CD34, CD117, myogenin, and DOG1 (discovered on GIST, also known as anoctamin 1), have also been proposed to play a significant role in identifying soft tissue tumors [27]. For myogenin, a transcription factor of myogenesis [31], its expression was absent in our case, and it has been reported to be specific to the pediatric malignancy, rhabdomyosarcoma [32]. In the case of CD34, it is a glycosylated transmembrane protein that was mainly expressed on hematopoietic and endothelial cells [33]. Its positive expression has been noted in cases of tumors with fibroblastic lesions, possibly owing to its expression on the mesenchymal progenitor cell, fibrocyte [34]; however, the expression was mostly negative in our study and other existing literature, as derived myofibroblastic cells do not express this protein [11, 22, 25, 35]. Last, for CD117 and DOG1, these two biomarkers were found in approximately 95% of GIST [36, 37], which may serve as the tool for differential diagnosis between GIST and IMT. In summary, a diagnostic flowchart was established (Figure 2). However, some of the markers were seldom stained in the cases in our study, and an adequate analysis was difficult to conduct because of the small sample size. Please refer to Table S1 for the marker results.

**Table 6:** IHC markers in the literature.

Literature	SMA	Desmin	CK	S‐100
Present study	29/39 (74.4%)	18/38 (47.3%)	7/26 (26.9%)	1/25 (4%)
Telugu et al. [11]	12/16 (75%)	5/11 (45.5%)	0/6 (0%)	N/A
Jung et al. [21]	9/10 (90%)	N/A	0/10 (0%)	5/10 (50%)
Salgueiredo‐Giudice et al. [22]	3/3 (100%)	0/3 (0%)	0/3 (0%)	0/3 (0%)
Wang et al. [23]	5/5 (100%)	5/5 (100%)	5/5 (100%)	0/5 (0%)
Qiu et al. [24]	23/24 (95.8%)	7/21 (33.3%)	3/22 (13.6%)	0/22 (0%)
Shi et al. [25]	N/A	1/5 (20%)	0/5 (0%)	0/5 (0%)
Da et al. [26]	4/5 (80%)	11/17 (64.7%)	8/13 (61.5%)	1/12 (8.3%)
Summary	85/92 (92.3%)	47/100 (47%)	23/90 (25.6%)	7/82 (8.5%)

*Note:* The denotation was (number of positive expression)/(number of stained cases).

Abbreviations: CK, cytokeratin; SMA, smooth muscle actin.

This study had some limitations. First, owing to the rarity of IMT, collecting a sufficient sample size was challenging. Only 48 cases could be gathered during the period of 21 years (2001–2022). Second, there is no consensus on the markers that should be stained for the diagnosis of IMT. Therefore, the number of markers analyzed was limited to those mentioned above (ALK, SMA, desmin, cytokeratin, S‐100 protein, myogenin, CD34, CD117, and DOG1). Third, it would be better if all patients underwent alternative detection measurements (e.g., FISH and NGS) to further confirm the IHC results. However, owing to the difficulty of assessing, such as cost, this was not feasible in our case.

In conclusion, we present real‐world data from 48 patients with IMT at various sites and discuss the results of IHC staining. Although the gold standard of diagnosis is histology and IHC staining, those with ALK(−) IMT still struggle with a definitive diagnosis. We hypothesized that ALK expression may vary with the site of origin based on the categories of visceral and nonvisceral organs. SMA was nearly always positive, whereas S‐100 protein, myogenin, CD34, CD117, and DOG1 were almost negative in all cases. For desmin and cytokeratin, the results differed from case to case and may not be used as determinant factors to diagnose such a disease. We hope that this investigation will be a cornerstone for further studies on the diagnosis of IMT in the absence of ALK rearrangements.

## Conflicts of Interest

The authors declare no conflicts of interest.

## Funding

This work was financially supported by grants from Linkou Chang Gung Memorial Hospital (Grants CMRPVVP0111 and CMRPVVQ0041 to Chiao‐En Wu) and the National Science and Technology Council (Grants 113‐2628‐B‐182‐001‐MY3 and 113‐2811‐B‐182‐024 to Chiao‐En Wu).

## Supporting Information

Additional supporting information can be found online in the Supporting Information section.

## Supporting information


**Supporting Information** The attached Table S1 lists the markers (e.g., CD21, CD35, and CD23) that were stained other than those mentioned in the article (ALK, SMA, desmin, cytokeratin, S‐100 protein, CD34, CD117, myogenin, and DOG1).

## Data Availability

All data generated or analyzed during the study are included in this article. Further inquiries can be directed to the corresponding author.
