# Supplementary material for: Clinicopathological Features and Immunochemical Staining of Inflammatory Myofibroblastic Tumor: A Retrospective Study of 48 Cases
Source: Anal Cell Pathol (Amst). 2025 Nov 18;2025:4948627. doi: 10.1155/ancp/4948627 (PMC12624268; doi:10.1155/ancp/4948627)
Supplement: Supplementary file 1 — Supporting Information The attached Table S1 lists the markers (e.g., CD21, CD35, and CD23) that were stained other than those mentioned in the article (ALK, SMA, desmin, cytokeratin, S‐100 protein, CD34, CD117, myogenin, and DOG1). [file ANCP-2025-4948627-s001.docx]

Supplementary data

| Supplementary table 1. Other IHC markers that were stained in less than half of patients. | | | | |
| --- | --- | --- | --- | --- |
| Markers | Positivity | | Negativity | |
| CD21 | 2 | | 18 | |
| CD35 | 2 | | 15 | |
| CD23 | 4 | | 9 | |
| HHF-35 | 4 | | 5 | |
| Calponin | 2 | | 5 | |
| BCL-2 | 1 | | 4 | |
| EMA | 2 | | 2 | |
| p63 | 1 | | 3 | |
| CD68 | 2 | | 2 | |
| Caldesmon | 0 | | 3 | |
| GATA3 | 0 | | 3 | |
| CD3 | 1 | | 2 | |
| CD1a | 0 | | 2 | |
| Estrogen receptor | 0 | | 2 | |
| Progesterone receptor | 0 | | 2 | |
| STAT6 | 0 | | 2 | |
| EBER | 0 | | 2 | |
| LMP1 | 0 | | 2 | |
| $\beta$-catenin | 1 | | 1 | |
| ROS1 | 0 | | 2 | |
| Pan-Trk | 0 | | 2 | |
| Podoplanin | 0 | | 2 | |
| MDM2 | 1 | | 1 | |
| CD10 | 0 | | 1 | |
| CD20 | 0 | | 1 | |
| CD30 | 0 | | 1 | |
| CD31 | 0 | | 1 | |
| CD56 | 0 | | 1 | |
| CD99 | 0 | | 1 | |
| TTF-1 | 0 | | 1 | |
| HMB45 | 0 | | 1 | |
| Melan-A | 0 | | 1 | |
| TLE1 | 0 | | 1 | |
| HER2 | 0 | | 1 | |
| HHV8 | 0 | | 1 | |
| SOX10 | 0 | | 1 | |
| Glypican 3 | 0 | | 1 | |
| CEA | 0 | | 1 | |
| Cyclin D1 | 0 | | 1 | |
| PAX-8 | 0 | | 1 | |
| IRF4/MUM1 | 0 | | 1 | |
| Chemokine ligand 13 | 0 | | 1 | |
| CDK4 | 0 | | 1 | |
| Calretinin | | 0 | | 1 |
| CD: cluster of differentiation, HHF-35: muscle actin specific monoclonal antibody, BCL-2: B cell lymphoma-2, EMA: epithelial membrane antigen, GATA3: GATA binding protein 3, STAT6: signal transducer and activator of transcription 6, EBER: Epstein-Barr virus encoded small RNAs, LMP1: latent membrane protein 1, ROS1: proto-oncogene tyrosine protein kinase 1, MDM2: mouse double minute 2, TTF-1: thyroid transcription factor 1, HMB45: human melanoma black 45, Melan-A: melanoma antigen recognized by T cells 1, TLE1: transducin-like enhancer protein 1, HER2: human epidermal growth factor receptor 2, HHV8: human herpesvirus 8, SOX10: SRY-Box transcription factor 10, CEA: carcinoma embryonic antigen, IRF4/MUM1: interferon regulatory factor 4 protein/multiple myeloma 1, CDK4: cyclin dependent kinase 4 | | | | |
